# Living in the vicinity of pesticide-treated crop fields: Exploring associated perceptions and psychological aspects in relation to self-reported and registry-based health symptoms

**DOI:** 10.1186/s40359-024-02162-1

**Published:** 2024-11-16

**Authors:** J Gerbecks, C Baliatsas, CJ Yzermans, M Simoes, A Huss, RA Verheij, MLA Dückers

**Affiliations:** 1 Netherlands Institute for Health Services Research (Nivel), Utrecht, 118-124, 3513 CR The Netherlands; 2 Institute for Risk Assessment Sciences (IRAS), Utrecht University, Utrecht, The Netherlands; 3 Tilburg School of Social and Behavioral Sciences (TRANZO), Tilburg University, Tilburg, The Netherlands; 4 Faculty of Behavioural and Social Sciences, University of Groningen, Groningen, The Netherlands; 5 ARQ National Psychotrauma Centre, Diemen, The Netherlands

**Keywords:** Environmental worry, Pesticides, Environmental hazards, Non-specific symptoms, Medically unexplained symptoms, Perceived exposure

## Abstract

**Background:**

Exposure to pesticides in the living environment can be associated with the prevalence of health symptoms. This study investigates associations between health symptoms among residents in areas with fruit crop fields where pesticides are applied, and psychological perceptions and attitudes about environmental aspects and exposures.

**Methods:**

A cross-sectional survey combined with routine primary care electronic health records (EHR) data was conducted in 2017 in rural areas of the Netherlands with high concentration of fruit crops (*n* = 3,321, aged ≥ 16 years). Individual exposure to pesticides was estimated using geocoded data on fruit crops around the home. Validated instruments were used to assess symptom report and psychological perceptions and attitudes. Annual prevalence of various health symptoms was derived from EHRs. Multilevel regression models were used to analyze associations between health symptoms (outcome), fruit crops, and multiple psychological perceptions and attitudes (confounders).

**Results:**

Living in the vicinity of fruit crop fields was generally not associated with self-reported symptom duration and general practitioner (GP) registered symptoms. For self-reported symptoms, symptom prevalence decreased when crop density within 250 m and 500 m from the home increased. No associations were found at other distances. Furthermore, higher levels of environmental worries, perceived exposure, and perceived sensitivity to pesticides and attribution of symptoms to environmental exposures were generally associated with a higher number of self-reported symptoms, and longer symptom duration. Symptoms reported to GPs were not associated with psychological perceptions and attitudes, except for perceived sensitivity to pesticides.

**Conclusion:**

Psychological perceptions and attitudes appear to be related to self-reported symptoms, but not to GP-registered symptoms, independent of the actual levels of exposure as measured by the size of the area of crop fields. Perceptions about environmental factors should be taken into account in environmental health risk assessment research when studying health symptoms.

**Supplementary Information:**

The online version contains supplementary material available at 10.1186/s40359-024-02162-1.

## Introduction

Pesticides are “chemical compounds that are used to kill pests, including insect, rodents, fungi and unwanted plants (weeds)” [1]. They are common practice in agriculture to preserve harvest. However, they can transfer from agricultural fields to homes through volatilization from soil or crops [2]. Several studies found pesticide concentration in house dust samples [3–6], measured up to 1,250 m from agricultural sites [5, 6], and pesticide concentrates were higher in houses located within 250 m to agricultural spray locations, compared to locations further away [3].

Despite pesticides serving a good purpose, they also do harm. Pesticides have been linked to various chronic diseases, such as cancer [7], atopic asthma [8], and Parkinson’s disease [9], and to the acute symptoms nausea, headache, and dizziness [10–12]. The vast majority of studies among residents living in the vicinity of pesticide treated lands focused on chronic and congenital conditions, and were performed in the United States [13–15].

Studies on acute health symptoms are more sparse, despite these symptoms being common in the general population. Although, from a medical perspective, such symptoms can be linked to chronic conditions as a precursor, indication, characteristic or effect, when general practitioners (GPs) in primary care cannot find a clinical explanation or fitting diagnosis, they will classify them as symptoms, instead of conditions or diseases. These symptoms can be described as ‘medically unexplained symptoms’, or ‘non-specific symptoms’ (NSS) [16, 17].

Since the etiology is often unknown, NSS are sometimes attributed to various environmental exposures that are tolerated by the vast majority of people and are below toxicologically harmful doses [18], such as electromagnetic fields [19, 20], noise [21, 22], odors [23, 24], and wind turbines [25]. Attribution of symptoms to (perceived) exposure to environmental hazards or to chronic conditions is complicated because it requires a careful objective weighing of information on factors (including confounders) typically unavailable to residents or healthcare professionals. Mofatt et al. [26] described ‘awareness bias in environmental health research’ as ‘the tendency to report more illness because of concerns arising from proximity to a hazard in the absence of a measurable biological effect’. Witthöft and Rubin [27] concluded that media reports about potential environmental hazards can increase experienced symptoms following (sham) exposure. While some studies highlighted the possible role of psychological perceptions and attitudes in symptom attribution processes, evidence at the population level remains relatively limited. Studies on the perceptions towards the use of pesticides focus mainly on farmers rather than residents [28], or examined different countries with different practices and regulation concerning pesticide use, and with different population densities in close vicinity to crop fields [29]. Köteles and Simor [30] and Petrie et al. [31] established a relation between modern health worries (MHW) and attribution of unexplained symptoms. Petrie et al. [31] concluded that higher levels of MHW were associated with higher number of symptoms being attributed to pesticide spraying. Baliatsas et al. [19] found that MHW are also associated with increased non-specific symptoms registered in primary care. These studies support the idea that psychological perceptions and attitudes regarding environmental exposure may have an influence on (perceived) health [32].

The aim of this study is to investigate the associations between health symptoms and (perceived) exposure to pesticides, combined with psychological perceptions and attitudes about environmental exposures in rural areas of the Netherlands. In rural areas in The Netherlands, about 18% of all people live within 250 m from crops [3]. A fair share of society thus is potentially exposed to higher levels of pesticides. Previous studies on pesticides and human health symptoms primarily relied on self-reported data. This study uses a combination of self-reported and GP-registered symptoms, enabling an examination of the attribution of symptoms to pesticide exposure in both datasets. The research question is: to what extent exposure to pesticides is related to health symptoms of residents, and what role do psychological perceptions and attitudes play in this association?

## Materials and methods

### Study design and participants

The current study was performed within the framework of the study ‘Health survey on people living in the direct vicinity of agricultural plots’ [33]. This study combines general practice data with questionnaire data. First, general practices located in fruit growing areas in the Netherlands were selected from the Netherlands Institute for Health Services Research (Nivel) Primary Care Database (PCD). The Nivel PCD meets the Dutch regulations on data protection and laws on use of health data for epidemiological research purposes (Dutch Civil Law, Article 7:458). Fruit growing areas were chosen because fruit crops represent a stable type of cultivation that does not considerably vary over time (Spearman rho correlation of 0.8–0.98) [33]. Eleven GP practices were included, in which about 56,000 patients were registered. A Trusted Third Party (TTP) (“Stichting Informatie Voorziening Zorg: IVZ”, Houten, The Netherlands) was involved in the process of sending questionnaires to residents enlisted as GP-patients, and processing the data, to ensure anonymity at all times. The TTP selected 12,000 potential survey participants, of which 3,853 filled out a questionnaire during the pesticide spraying season in the summer of 2017 (response rate 32%). Only patients that were registered between 2014 and 2016 at those practices, and aged 16 years or older were eligible to fill in the questionnaire. At most one person per household could participate. Questionnaire data were merged with available symptom data from electronic health records (EHRs) and proxies of pesticide exposure for the same individuals (see Sect. 2.2.1–2.2.3). After merging the different datasets, combined EHR and survey data for 3,321 persons were available for analysis. Data is available on request due to privacy/ethical reasons. A flow chart of the data collection process is shown as Supplement 1.

### Measures

#### Assessment of Crop Density

**Fig. 1:**
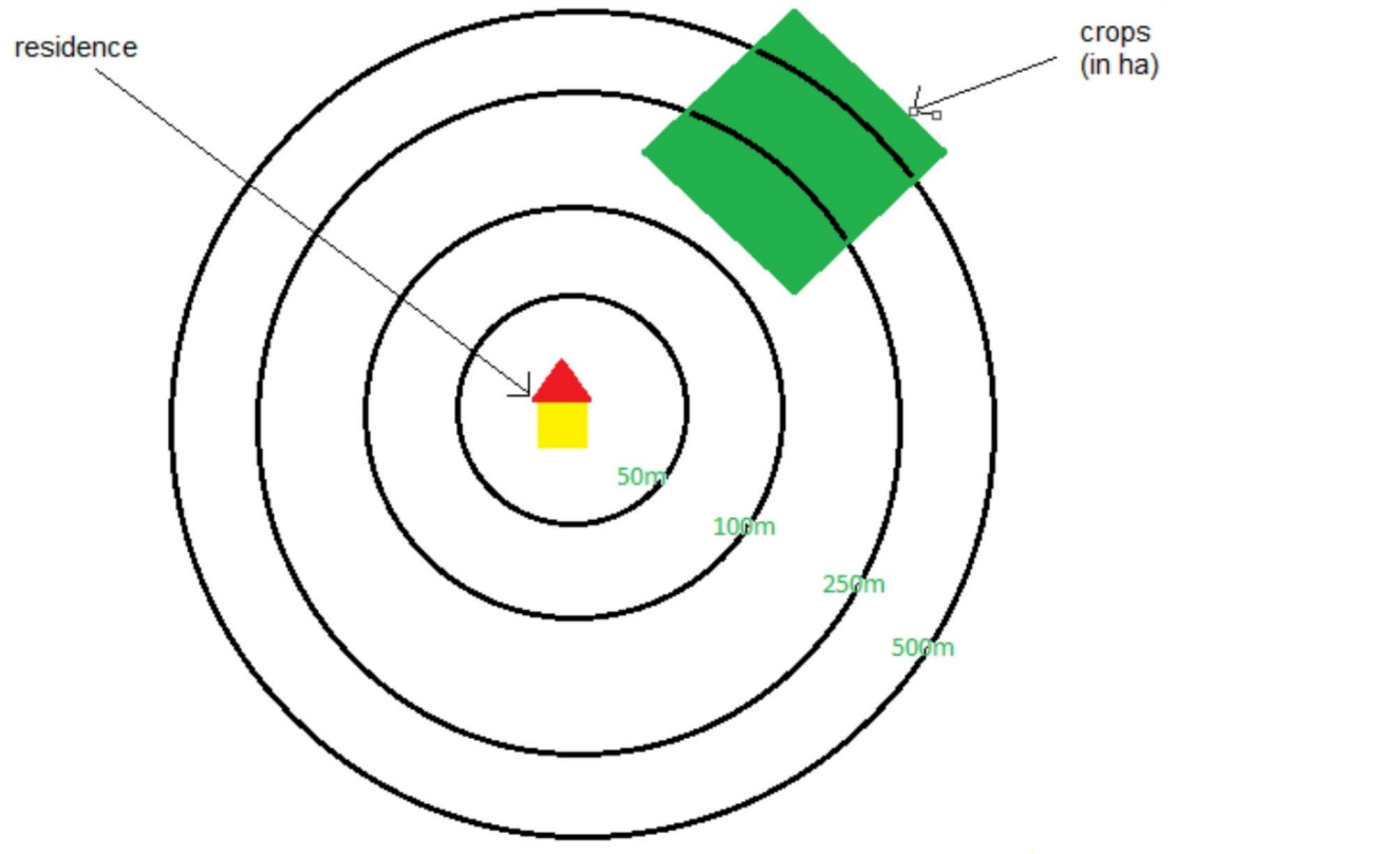
Visualization of a buffer zone; fruit crops are present within 250 and 500 m from residence

Fruit crop area was estimated using spatial data (2014–2016) from the Basic Registration of Crop Parcels ‘Basisregistratie Gewaspercelen’, BRP 2016 [33, 34]. Buffer exposure zones were calculated as a proxy for exposure to pesticides in the residential environment. The square area (in hectares) of fruit crops within a buffer surrounding one’s house (0–50 m, 0–100 m, 0–250 m, and 0–500 m) was estimated. These buffers were treated as continuous variables. Participants living outside a given buffer/radius were coded as “0”, while values larger than 0 indicate the hectares of crop fields within the buffer. For instance, a value of 0.05 for a 0–250 m buffer means that there are 0.05 hectares of fruit crop in the 0–250 m squared buffer zone around the address (Fig. 1).

#### Self-reported outcome measures

The Symptoms and Perceptions (SaP) questionnaire [35] was used to assess self-reported symptoms. For 28 health symptoms, the respondents were asked whether or not they experienced a given symptom in the previous month, for how long they had been experiencing that symptom, and whether they consulted their GP with that symptom. Two variables for self-reported symptoms were constructed; one for the sum score of symptoms, and one for the total duration of these symptoms. A higher score on these variables indicates increased symptom report and longer duration. The list of symptoms that the survey asked about, is displayed in the Supplement 2.

#### Routine GP electronic health records data

In the Netherlands, each inhabitant is obligatory enrolled in just one general practice, ensuring comprehensive health records. GPs are the first to contact with a health problem. Because GPs systematically register all reported health problems, there is no recall bias. GP EHRs can therefore be seen as an effective way to assess population health without asking patients themselves.

Morbidity is registered according to the International Classification of Primary Care (ICPC) [36]. Each health symptom can be classified with both a letter (representing the organ system in which the symptom occurred) and a number (describing a specific symptom). The ICPC-codes used for this study can be found in the Supplement 2. ICPC codes from EHRs were matched to the corresponding health symptoms in the questionnaire. In some cases, symptom clusters were used, similar to the clusters in Simoes et al. [33] (see Supplement 2).

#### Psychological aspects

Perceived exposure to pesticides was measured in the survey by the question “To what extent do you think you are exposed to pesticides?”. Respondents were asked to answer this question for four situations − 1) at home, 2) at work, 3) outdoors, and 4) in food - using a 10-point scale, adapted from Baliatsas et al. [19]. The items were combined into a single variable, demonstrating a satisfactory level of internal consistency with a Cronbach’s Alpha coefficient of 0.78. Respondents that answered every question with ‘no perceived exposure’ (and thus scored 0) were used as reference category (13.4% of the respondents). We determined a score of average 0 to 2 on four items to be ‘moderate perceived exposure’ (37% of the respondents), a score of 2 to 4 ‘high perceived exposure’ (21.5% of the respondents), and very high perceived exposure was assigned to everyone scoring between 4 and 10 (9.1% of the respondents). 19% of the respondents did not answer this question.

General environmental worries were measured using 14 items from the Modern Health Worry (MHW) questionnaire [37], covering “people’s concerns about possible harmful effects of modern technologies”, based on a five-point scale (‘not worried at all’ to ‘extremely worried’) [35]. An exploratory factor analysis showed twelve of the fourteen items to be eligible to measure one factor: air pollution, exhaust gases, climate change/greenhouse effect, use of a pest control spray, genetically engineered foods, food additives, pesticides in foods, antibiotics in foods, polluted drinking water, toxic chemicals in household products, bacteria in air conditioning or hot water systems (e.g. legionella), and bacteria resistant to medication. These twelve items were added, and this score was dichotomized at the median in order to categorize participants with little environmental worry and the ones with higher levels of environmental worries. The two items not eligible for this factor were omitted.

Attribution of symptoms to environmental exposures was assessed by asking respondents about their most important (self-reported) symptom and its (presumed) cause (based on a selection of various possible causal factors [38]). These causes were categorized into two groups: ‘environmental issues’ and ‘unknown/other cause’ (reference category).

A single item to measure subjective sensitivity to pesticides was used (“I am sensitive to pesticides”), formatted on a five-point scale, which was part of a broader list assessing sensitivity to diverse environmental stressors, adapted from Stansfeld et al. [39] and Baliatsas et al. [40]. Participants that answered ‘completely agree’ or ‘fairly agree’ were considered ‘sensitive’, while all others were included in the reference category.

### Statistical analyses

Multilevel regression analyses were conducted, accounting for the hierarchical data structure (patients nested within general practices). Depending on the outcome variable, either multilevel logistic regression or multilevel negative binomial regression analysis was used.

Analyses concerned the following associations: (1) fruit crop density (buffers) and symptom scores (both number of symptoms and duration), (2) psychological perceptions and attitudes (high general environmental worry, perceived exposure to pesticides, presumed sensitivity to pesticides, and attribution of NSS to environmental exposures), and both symptom scores. Furthermore, a number of additional and sensitivity analyses were performed: associations between the investigated psychological aspects (perceived exposure, environmental worry, environmental sensitivity and causal attribution) and individual NSS (instead of symptom scores) were assessed. In addition, analyses on perceived exposure and general environmental worry were repeated as continuous variables (instead of categorical, as included in the main text), to examine whether a different variable operationalization impacted the reported associations. We also executed the analyses without accounting for pesticide use at work, to see whether we overcontrolled the analyses possibly for a large group of fruit farmers in our dataset.

All analyses were adjusted for age, gender, educational level, financial status, ethnicity, smoking habits, BMI, and self-reported pesticide exposure at work. The analyses on the psychological variables and the sensitivity analyses were additionally corrected for the buffers, as the most relevant proxy of pesticide exposure in the residential environment [33].

For each association, odds ratios (OR) or incidence rate ratios (IRR), and 95% confidence intervals (CI) were calculated. N per analysis can vary, based on the lowest N for the variables included in that analysis. For the N per variable, see Table 1. Analyses were performed with STATA version 15.0 (StataCorp LP, College Station, TX, USA).

**Table 1:** Overview of demographic, residential, lifestyle and symptom characteristics (*N* = 3,321)

Demographic characteristics		
% Age (*N* = 3,296)		
16–24	4.34	
25–44	16.99	
45–64	38.08	
65–74	24.81	
75+	15.78	
Mean age (SD) (*N* = 3,296)	58.03	(17.06)
% Female gender (*N* = 3,306)	56.61	
% Educational level (*N* = 3,291)		
No education	1.52	
Primary school	6.96	
Pre-vocational education	17.78	
Secondary education	14.89	
Secondary vocational education	20.18	
Secondary higher education	7.69	
Higher education	23.15	
University	7.84	
% Financial situation (*N* = 3,215)		
I have to overdraw/am in the red	2.36	
I have to use up savings (partly)	7.84	
I can just get by	20.81	
I can save a little money	51.07	
I can save a lot of money	17.92	
% Foreign background (*N* = 3,286)	3.37	
Mean Body Mass Index (SD) (*N* = 3,244)	25.99	(4.58)
**Residential characteristics**		
% Degree of urbanization (*N* = 3,321)		
Extremely urbanized	0.03	
Strongly urbanized	3.64	
Moderately urbanized	9.55	
Hardly urbanized	34.96	
Not urbanized	51.82	
**Lifestyle characteristics**		
% Smoking habits (*N* = 3,253)		
Never	45.56	
In the past	41.07	
Yes, currently	13.37	
% Use of pesticides at work (*N* = 3,094)	5.20	
**Psychological Perceptions and Attitudes**		
High general environmental worry (%) (*N* = 3,071)	43.63	
Perceived exposure to pesticides (%) (*N* = 2,691)		
No perceived exposure	16.54	
Low perceived exposure	45.67	
Moderate perceived exposure	26.53	
High perceived exposure	11.26	
% Perceived sensitivity to pesticides (*N* = 3,214)	16.96	
% attribution of NSS to environmental exposures (*N* = 3,321)	1.87	
**Symptom characteristics**		
Number of NSS mean score (SD) (*N* = 2,598)	4.57	(4.32)
Duration of NSS mean score (SD) (*N* = 2,574)	12.69	(14.23)
GP-registered NSS mean score (SD) (*N* = 3,321)	0.43	(0.79)

Supplement 1. Study data collection process

## Results

### Descriptive results and non-response

**Fig. 2:**
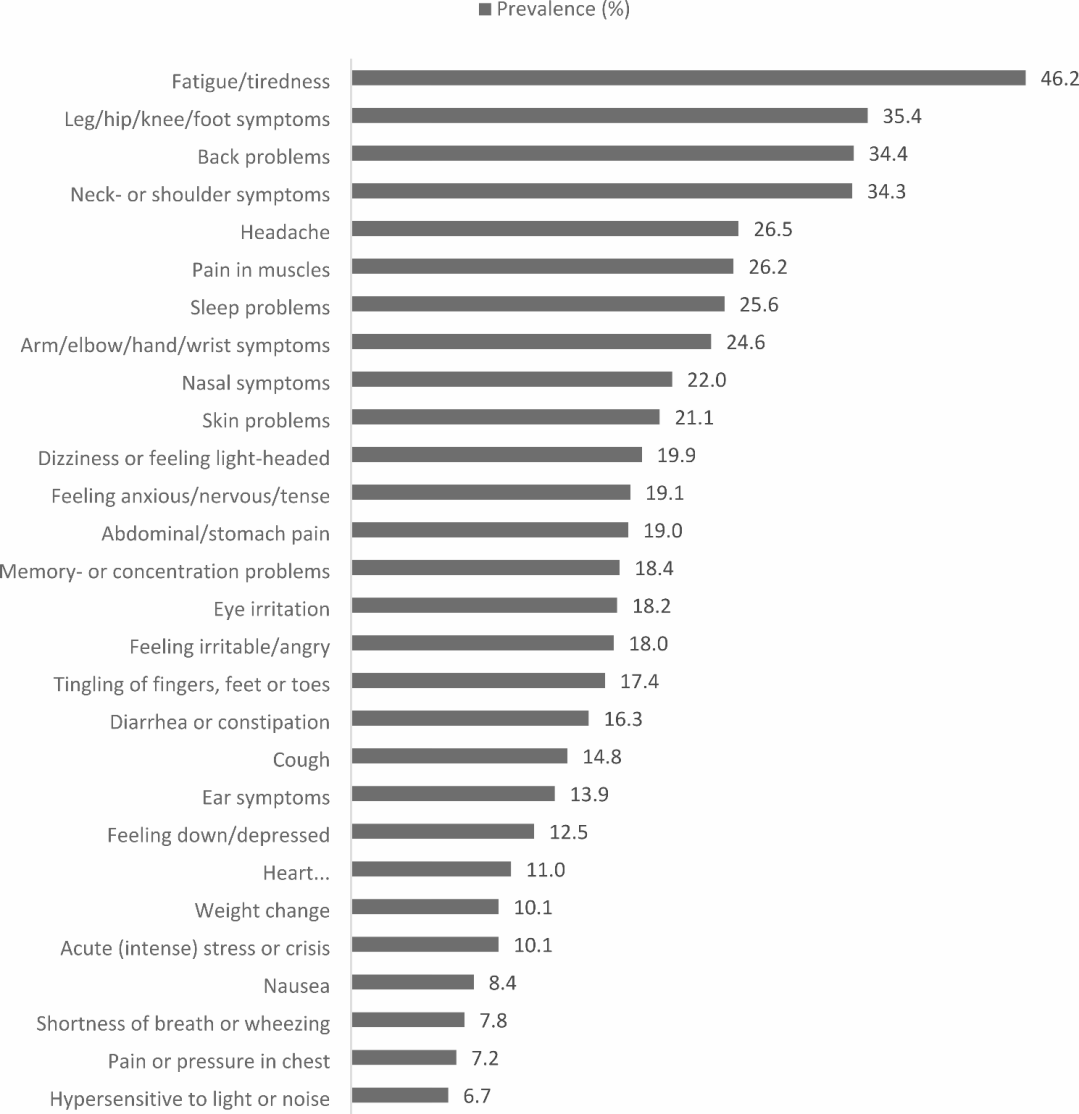
Percentage of the study population with a self-reported symptom “in the past month”

**Fig. 3:**
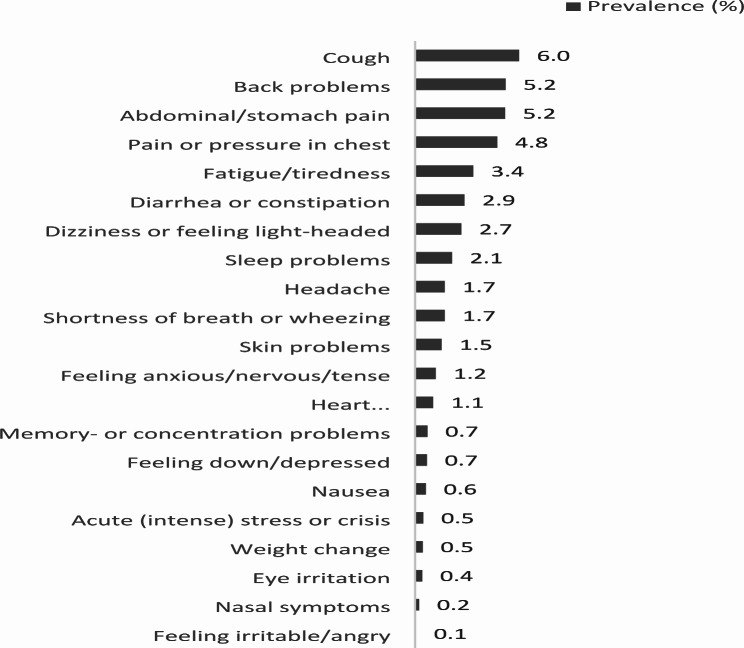
One-year prevalence of GP-registered symptoms in the study population

As a non-response analysis, we compared the demographic characteristics of survey responders to the total pool of potential survey participants. Based on the year 2016 in the EHRs, survey respondents were significantly (*p* < 0.01) older and more often women, compared to the total practice population (48 vs. 58 years, and 50 vs. 57% respectively). Table 1 gives an overview of demographic, residential, lifestyle and symptom characteristics. Figure 2 shows the prevalence of the self-reported NSS. People that did not report presence of a certain symptom were included as if they had answered ‘no’ to the question whether they experienced that symptom. Figure 3 shows the prevalence of the GP-registered NSS. The one-year prevalence of eight symptoms was below 1%.

The correlations between perceived exposure and geocoded exposure buffers are shown in Table 2. No significant association between perceived exposure to pesticides and actual exposure has been found: correlation was low and none of the associations was higher than 0.10.

**Table 2:** Correlations between perceived exposure to pesticides and area of fruit crops in a buffer

	Buffers					
	0–50 m	0–100 m	0–250 m	0–500 m		
No exposure	− 0.04	− 0.04	− 0.01	− 0.05		
Little to average exposure	− 0.00	− 0.01	− 0.01	− 0.02		
Above average exposure	− 0.01	− 0.02	− 0.03	0.00		
High levels of exposure	0.07	0.10	0.07	0.08		

Based on chi square tests, correlation between perceived exposure as one categorical variable and each of the buffers are non-significant as well.

#### Associations between fruit crop buffers and symptom scores

Table 3 shows the Incidence Rate Ratios (IRRs) between crop density within a designated buffer, and the self-reported and GP-registered symptoms.

**Table 3:** Association^a^ between fruit crop density buffers and NSS in self-reported and GP-registered prevalence (CI 95%)

	Number of self-reported NSS						Duration of self-reported NSS								Number of GP registered NSS					
	IRR	CI					IRR		CI						IRR			CI		
0–50 m	0.71	(0.42	-		1.19)		0.78		(0.42		-		1.43)		0.43			(0.13	-	1.34)
0–100 m	0.92	(0.81	-		1.02)		0.94		(0.82		-		1.08)		0.87			(0.69	-	1.10)
0–250 m	**0.97**	**(0.94**	**-**		**1.00)** ^**b**^		0.97		(0.94		-		1.01)		0.97			(0.92	-	1.03)
0–500 m	**0.995**	**(0.99**	**-**		**1.00)** ^**c**^		1.00		(0.99		-		1.00)		1.00			(0.99	-	1.00)

Abbreviations: IRR; Incidence Rate Ratios; CI, confidence intervals

^a^ Adjusted for gender, age, ethnicity, education, financial situation, BMI, smoking behavior, and pesticide use at work

* *p* < 0.05

** *p* < 0.01

*** *p* < 0.001

^b^ IRR (0.937 − 0.995), *p* = 0.023; ^c^ IRR (0.991 − 0.9994), *p* = 0.028

Analyses showed two significant, albeit small, associations between higher fruit crop density nearby and higher self-reported symptoms. For longer symptom duration and GP registered symptoms, no significant associations were found. Within the buffers 0–250 m and 0–500 m, chances of experiencing more symptoms were significantly smaller as crop density within the buffer increased.

#### Associations between psychological perceptions and attitudes and symptom scores

**Fig. 4:**
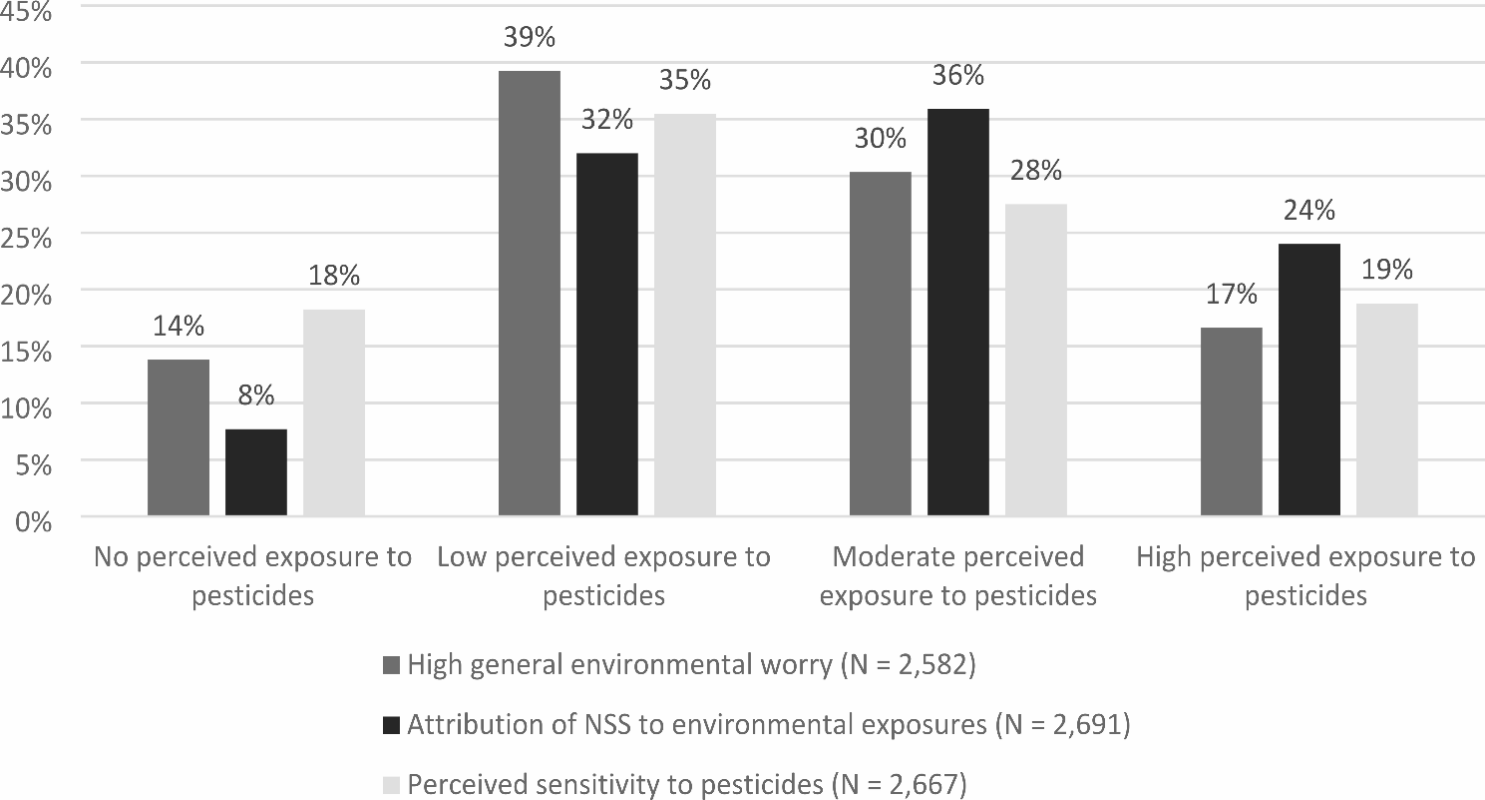
Percentage of psychological aspect in each of the perceived exposure groups

Before we conducted the regression analyses on the psychological perceptions and attitudes, we considered the distribution of these attitudes amongst the different perceived exposure categories. Figure 4 shows percentages of participants with respectively high general environmental worry, people that attribute their NSS to environmental exposures, and people with perceived sensitivity to pesticides within any of the exposure groups.

It showed that individuals reporting low and moderate levels of perceived exposure to pesticides had the highest levels of environmental worry, perceived sensitivity to pesticides, and mostly mentioned an environmental exposure as the cause of their most important symptoms. More specifically, more than half (60%) of the people who attributed their symptoms to environmental exposures also thought that their level of exposure to pesticides was moderate or high. About half of the people (47%) with high general environmental worry considered themselves to be moderately or highly exposed to pesticides. Among the individuals sensitive to pesticides, more than half thought they were not or only low exposed to pesticides.

#### Associations between psychological factors and symptom scores

Table 4 shows the Incidence Rate Ratios (IRRs) the various psychological factors, and the self-reported and GP-registered symptoms.

**Table 4:** Associations^a^ between psychological perceptions and attitudes and symptom reporting (CI 95%)

	Number of self-reported NSS					Duration of self-reported NSS								Number of GP registered NSS				
	IRR	CI				IRR		CI						IRR	CI			
High general environmental worry	**1.14**	**(1.06**	**-**	1.23)**		**1.16**		**(1.05**		**-**	1.28)**			1.05	(0.91		-	1.20)
Perceived pesticide exposure (No exposure = reference category)																		
Low perceived pest. exposure	**0.86**	**(0.79**	**-**	0.93)***		**0.82**		**(0.75**		**-**	0.90)***			0.99	(0.85		-	1.14)
Moderate perc. pest. exposure	**1.16**	**(1.07**	**-**	1.27)***		**1.23**		**(1.11**		**-**	1.37)***			0.91	(0.77		-	1.08)
High perceived pest. exposure	**1.30**	**(1.15**	**-**	1.47)***		**1.31**		**(1.13**		**-**	1.53)***			0.98	(0.77		-	1.24)
Perceived sensitivity to pesticides	**1.29**	**(1.16**	**-**	1.44)***		**1.32**		**(1.16**		**-**	1.51)***			**1.21**	**(1.02**		**-**	1.43)*
Attribution of NSS to environmental exposures	**1.66**	**(1.25**	**-**	2.20)***		**1.61**		**(1.11**		**-**	2.31)*			1.31	(0.84		-	2.06)

Abbreviations: IRR; Incidence Rate Ratios; CI, confidence intervals

^a^ Adjusted for gender, age, ethnicity, education, financial situation, BMI, smoking habits, pesticide use at work, and the crop density buffer (0–250 m)

* *p* < 0.05; ** *p* < 0.01; *** *p* < 0.001

Multiple statistically significant associations appeared between the psychological perceptions and attitudes and different symptoms as indicated in Table 4. The number of self-reported symptoms and the duration of these symptoms was significantly higher for participants who worried more about the possible risks of environmental exposures on their health, and the ones who considered themselves being moderately exposed or highly exposed to pesticides. In addition, higher levels of perceived sensitivity to pesticides, and attribution of symptoms to environmental causes were also associated with increased symptom report and duration. On the contrary, participants who perceived low exposure also experienced fewer NSS, and experienced their NSS with a shorter duration than participants who perceive themselves as unexposed. For the GP registered number of symptoms, there was a higher chance at more symptoms for participants with higher levels of perceived sensitivity to pesticides.

#### Sensitivity analyses

Sensitivity analyses were performed to assure that the results in this study are not biased by the construction of the variables. The results of the sensitivity analyses were in line with the main analyses: when the individual symptoms (instead of total scores) were analyzed, many of the self-reported symptoms were significantly (*p* < 0.05) associated with perceived exposure, environmental worries, environmental sensitivity and causal attribution. Similar to the main analyses, generally no significant associations between the psychological factors and the GP-registered symptoms were observed. Also, excluding ‘pesticide use at work’ from the analyses did not alter the results. Moreover, all reported associations remained statistically significant when perceived exposure and environmental worries were analyzed as continuous variables (data not shown).

## Discussion

This study aimed to investigate the associations between crop field sizes, environmental perceptions, and health symptoms, considering various lifestyle and socio-demographic factors. Higher fruit crop density was barely associated with health symptoms. Significant associations were very small, and indicated a different relation that one might expect: more fruit crops were associated with less self-reported health symptoms. High general environmental worry, moderate to high perceived exposure to pesticides, and attribution of symptoms to environmental exposures were associated with higher number and longer duration of self-reported NSS. For GP-registered symptoms, only those with higher perceived exposure experienced significantly more symptoms. In addition, higher levels of perceived sensitivity to pesticides were associated with higher number of both self-reported and GP-registered NSS, and longer duration of self-reported NSS.

In general, it seems that people experience symptoms and also attribute these symptoms to environmental exposure, but they do not turn to their general practitioner with these symptoms. Our findings on the self-reported symptoms fit the attribution narrative of Mofatt et al. [26] and Witthöft et al. [27]. The findings also modestly support attempts to unravel the mechanism through which environmental stress produces elevated symptom levels through cognitive and behavioral models (see for instance Lazarus and Folkman [41], and Rief and Broadbent [42]). However, it does not explain decreased symptom-reporting with increasing density of fruit crops. A number of studies have shown that health symptoms can develop by short-term exposure to high levels of pesticides, but also by long-term exposure to low levels of pesticides [11, 43]. This emphasizes the need to consider psychological factors in environmental health risk assessments, to understand determinants of symptom reporting. It is a challenge to explain the possible impact of perceptions and attitudes. Those perceptions and attitudes do not always correspond with the actual presence of an environmental source that is (perceived as) a threat.

Previous studies in this field mainly focused on chronic diseases [7, 9], focused on farmers [7], or studied different countries with different regulation concerning pesticide use [29], which limits the comparability of findings. While some studies highlighted the possible role of perceptions and attitudes in symptom attribution processes, evidence at the population level remained relatively limited. A study in the Netherlands [44] found no statistically significant association between living near pesticide-treated land and psychological distress and self-perceived health. However, it did not explore potential determinants of perceived health. Several other studies have shown that in different contexts, psychological factors such as worries, perceptions and attitudes may play a role in (perceived) health [19, 28–31, 45]. Nevertheless, the majority of those studies did not account for the possible impact of actual exposure proxies.

A strength of the current study is that self-reported and GP-registered symptoms were evaluated in relation to environment-related perceptions, worries and attitudes, while also considering psychological variables and socio-demographic characteristics. We used a large sample from an area with a high concentration of fruit crops, based on objective estimates, and conducted the survey during the spraying season of 2017. This ensured that any NSS reported could be associated to the analyzed crop fields, as the self-reported symptoms were referring to the “last month”.

In the survey, questions regarding ambient environment and associated exposures were asked after the questions on health outcomes. By including symptoms presented to GPs, we were able to assess NSS based on the clinical assessment of a medical doctor. This is less susceptible to reporting bias compared to self-reported symptoms. We made use of a database and classification system with established reliability [36, 46].

A limitation of this study is that we could not include information about actual pesticide use/application, but rather concentration of crops in the home vicinity as a proxy for pesticide exposure. Nevertheless, validation studies have confirmed that the current approach provides reliable estimates of pesticide concentration in those areas [47]. It is feasible that rural populations live closer to other types of cultivation as well, but the results of this study are specifically focused on fruit crops. Therefore, none of the results can or should be generalized to other types of cultivation.

Definitely a strong feature of this study, is the strength of the GP registrations in the Netherlands. GPs register all encounters systematically, and all residents in the Netherlands are registered at one general practice. This minimizes chances of recall bias and selection bias. The risk of outcome misclassification is low. The combination of GP data and a health survey allows for exploration of associations between individual perceptions and an objective registration of health problems.

## Conclusion

Perceived exposure, environmental worries, causal attribution and perceived environmental sensitivity were significantly associated with more and longer-lasting self-reported symptoms, but not with GP-registered symptoms. This suggests a potential attribution of health symptoms to pesticide exposure. These results highlight the importance of considering individuals’ perceptions of environmental factors into health risk assessment research. Future studies should further explore the complex relationship between perceptions towards environmental risks, and objective health outcomes, to enhance our understanding of how environmental exposures relate to health symptoms.

## Electronic supplementary material

Below is the link to the electronic supplementary material.

Supplementary Material 1

Supplementary Material 2

## Data Availability

The datasets generated and/or analyzed during the current study are not publicly available due to privacy protection of the participants. The study’s privacy regulations state that only specifically designated researchers from Nivel have access to the study database. Sharing an anonymized and de-identified dataset is not possible as it would still contain Electronic Health Records as well as other personal data of participants, which could potentially lead to the identification of subjects. Researchers may contact the data access committee through Remco Coppen, PhD, LLM (r.coppen@nivel.nl) or non-personal departmental email (Nivel: zorgregistraties@nivel.nl) or contact Christos Baliatsas, PhD (c.baliatsas@nivel.nl).
