# Supplementary figures and images for: Living in the vicinity of pesticide-treated crop fields: Exploring associated perceptions and psychological aspects in relation to self-reported and registry-based health symptoms
Source: BMC Psychol. 2024 Nov 16;12:669. doi: 10.1186/s40359-024-02162-1 (PMC11569595; doi:10.1186/s40359-024-02162-1)

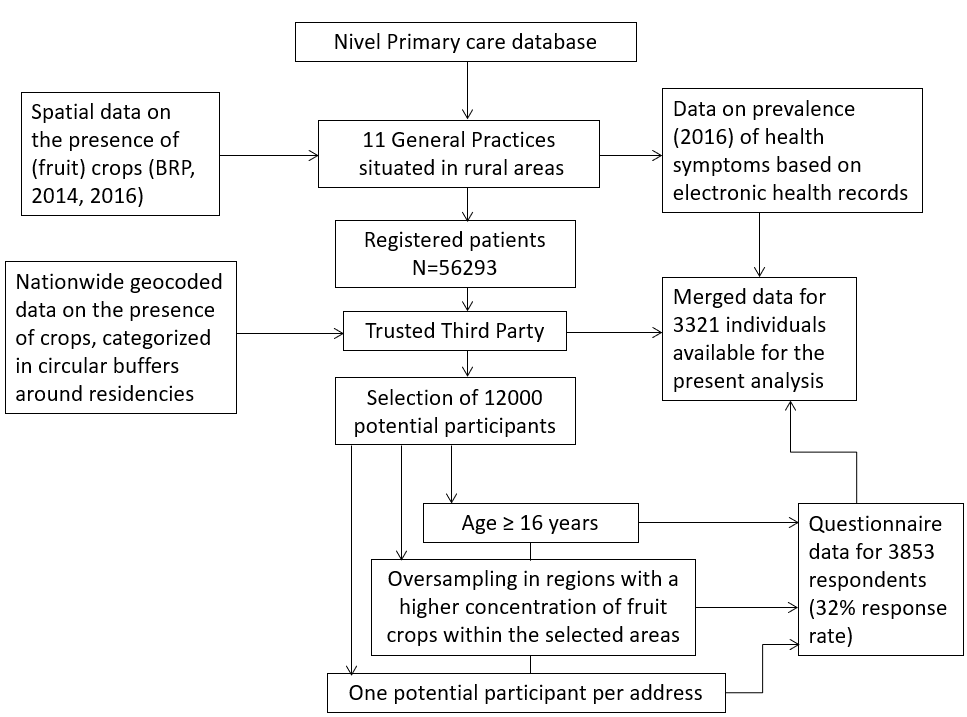


Figure 1. Study data collection process

Supplement: Supplementary file 1 — Supplementary Material 1 [file 40359_2024_2162_MOESM1_ESM.docx]
