# Supplementary material for: Living in the vicinity of pesticide-treated crop fields: Exploring associated perceptions and psychological aspects in relation to self-reported and registry-based health symptoms
Source: BMC Psychol. 2024 Nov 16;12:669. doi: 10.1186/s40359-024-02162-1 (PMC11569595; doi:10.1186/s40359-024-02162-1)
Supplement: Supplementary file 2 — Supplementary Material 2 [file 40359_2024_2162_MOESM2_ESM.docx]

**Appendix A**

| ICPC codes used for the symptom clusters | | | | |
| --- | --- | --- | --- | --- |
| Fatigue/tiredness | A04 |  |  |  |
| Abdominal/stomach pain | D01 | D02 | D06 |  |
| Nausea | D09 |  |  |  |
| Diarrhea or constipation | D11 | D12 |  |  |
| Eye irritation | F01 | F02 |  |  |
| Ear symptoms | H01 | H02 | H03 |  |
| Heart palpitations/awareness | K04 |  |  |  |
| Neck- or shoulder symptoms | L01 | L08 |  |  |
| Back problems | L02 | L03 |  |  |
| Pain or pressure in chest | L04 | K01 | K02 | K03 |
| Arm/elbow/hand/wrist symptoms | L09 | L10 | L11 | L12 |
| Leg/hip/knee/foot symptoms | L13 | L14 | L15 | L16 |
| Pain in muscles | L18 |  |  |  |
| Headache | N01 | N02 |  |  |
| Tingling of fingers, feet or toes | N05 |  |  |  |
| Dizziness or feeling light-headed | N17 |  |  |  |
| Feelings anxious/nervous/tense | P01 |  |  |  |
| Feeling down/depressed | P03 |  |  |  |
| Acute (intense) stress or crisis | P02 |  |  |  |
| Feeling irritable/angry | P04 |  |  |  |
| Memory- or concentration problems | P20 |  |  |  |
| Sleep problems | P06 |  |  |  |
| Shortness of breath or wheezing | R02 | R03 | R04 | R29 |
| Cough | R05 |  |  |  |
| Nasal symptoms | R06 | R07 | R08 |  |
| Skin problems | S01 | S06 | S07 |  |
| Weight change | T07 | T08 |  |  |
